# Multilocus sequence typing analysis of *Candida africana* from vulvovaginal candidiasis

**DOI:** 10.1186/s12879-019-4071-7

**Published:** 2019-05-22

**Authors:** Y. X. Zhu, Y. Shi, S. R. Fan, X. P. Liu, J. Yang, S. L. Zhong

**Affiliations:** 1 Department of Obstetrics and Gynecology, Peking University Shenzhen Hospital, Shenzhen, China; 2 Clinical College of Peking University Shenzhen Hospital, Anhui Medical University, Hefei, Anhui China; 3 Shenzhen Key Laboratory on Technology for Early Diagnosis of Major Gynecological Diseases, Shenzhen, China; 4 Department of Laboratory Science, Peking University Shenzhen Hospital, Shenzhen, China

**Keywords:** Vulvovaginal candidiasis, *Candida africana*, Multilocus sequence typing, Susceptibilities, Biofilm

## Abstract

**Background:**

*Candida africana* is distributed worldwide and colonized in human genitalia and cause mainly vulvovaginal candidiasis (VVC). We report the multilocus sequence typing (MLST) analysis of *C. africana* from VVC.

**Methods:**

MLST analysis of 43 strains of *C. africana,* which were isolated from vaginal specimens of patients with VVC, was performed. The enzymatic activity of phospholipase, esterase and haemolysis enzyme production was evaluated.The level of virulent genes and resistant genes mRNA expression was determined by using real-time PCR. Antifungal susceptibilities of the isolates were assayed by using the broth microdilution method. The statistical of the results was determined by the T test and Pearson chi-squared test.

**Results:**

The MLST analysis revealed a substantial degree of genetic homogeneity. The DST782 and DST182 were the main MLST genotypes in *C. africana.* All the patients were symptomatic and with a high mycological cure rate when treated with commonly used antifungal agents.There were statistically significant differences in biofilm formation and phospholipase activity between *C. africana* and *C.albicans*. The level of virulent genes and resistant genes mRNA expression was higher in fluconazole-resistant strains. All *C. africana* isolates were susceptible to fluconazole, itraconazole, voriconazole, caspofungin, and micafungin. These isolates also exhibited low MICs to amphotericin B, flucytosine, and posaconazole.

**Conclusions:**

*Candida africana* appear to be with a low level of sequence variation in MLST loci. *Candida africana*, a lower virulence candida, is susceptible to commonly used antifungal agents.

This paper was presented at the conference of 8th Trend in Medical Mycology (6–9 October 2017, Belgrade, Serbia) and was published on conference abstract.

**Electronic supplementary material:**

The online version of this article (10.1186/s12879-019-4071-7) contains supplementary material, which is available to authorized users.

## Background

*Candida africana* was isolated, for the first time, in 1995 in Madagascar, Africa and afterwards proposed as new Candida species phylogenetically closely related to *C. albicans* [1]. The isolates assigned to this group were originally proposed as representatives of a new species rather than a biovariant of *C. albicans*. And it shows remarkable differences if compared to *C. albicans* such as the taxonomic status. It has been reported that *C. africana* showed poor adhesion ability to human Hela cells, absence of biofilm formation, a notable low level of filamentation and significantly less pathogenic than *C. albicans* in the galleria mellonella insect systemic infection model [2–4]. *Candida africana* represents the phenotypic variation occurring in *C. albicans* [5]. *Candida africana* colonized mainly in human genitalia and cause vaginal infections [6, 7]. Its distribution appears to be worldwide [5–12]. The purpose of this study was to explore multilocus sequence typing (MLST) analysis of *C. africana* from vulvovaginal candidiasis and its relevance to clinical characteristics, virulence, pathogenicity, and antifungal profiles.

## Methods

### Fungal isolates and genotyping

The isolates were from vaginal specimens of patients with VVC and vaginal samples of pregnant women. Genomic DNA was extracted by using the E.Z.N.A™ Yeast DNA Kit (OMEGA, USA) according to the manufacturer’s instructions *HWP1* gene (primers CR-f 5-GCTAC CACTTCAGAATCATCATC-3 and CR-r 5-GCAC CTTCAGTCGTAGAGACG-3) amplification was performed and the PCR products was electrophoresed in 1.2% agarose gel in TBE buffer to distinguish *C. albicans*, *C. dubliniensis*, and *C. africana* on the basis of the distinct size of the amplicons as previously described [9, 13] (Additional file 6: Figure S1).

The ABC genotype and mating-type was determined by using previously designed primers and experimental conditions by McCullough et al. [14] and Hull et al. [15]. Multilocus sequence typing for *C. africana* was performed on the basis of a previously published consensus set of seven housekeeping gene loci for *C. albicans*: *CaAAT1a*, *CaACC1*, *CaADP1*, *CaMPIb*, *CaSYA1*, *CaVPS13* and *CaZWF1b* [16]. ALL seven loci were sequenced in both the forward and reverse directions and sequence data were assembled using ContigExpress software and checked manually for heterozygous polymorphisms. Allele numbers of each locus was determined by inputting the sequences in the *C.albicans* MLST database (http://calbicans.mlst.net) and diploid sequence types (DSTs) for each isolate was determined by the composite profile of all seven allele numbers. The MLST results of seven sequenced loci were concatenated into a single sequence to phylogenetic analysis by unweighted-pair group method using average linkages (UPGMA), determined by *P* distances using MEGA 6 software (http://www.megasoftware.net/).

*Candida albicans* SC5314 and *Candida albicans* ATCC90028 were used as reference strains.

### Quantitative real-time PCR

Quantitative real-time PCR analysis of following genes: *ALS1–7*, *ALS9*, *HWP1*, *SAP1-SAP10*, *PLB1–3*, *PLB5*, *CDR1*, *CDR2*, *MDR1*, *ERG11* and *HXK1* were performed as described by previous publications [17, 18]. Each experiment was carried out with ACT1 as the housekeeping gene. Data for each target gene were calculated as fold change in comparison with the reference gene ACT1 using the ΔΔCt quantification method. The primers used for quantitative real-time PCR analysis are shown in Additional file 1: Table S1.

### Sequencing of the *HXK1* gene

The DNA region of the *HXK1* gene carrying the mutation was amplified by or using the primer pair described by Felice et al. [19]. The amplified fragment was sequenced in both directions and sequence data were assembled using ContigExpress and compared with the corresponding wild-type *C. albicans* and *C. africana* reference strains respectively.

### Extracellular enzymes assays

The activity of phospholipase, esterase and haemolysis enzyme production was evaluated by or using plate assays methodology described by Pakshir et al. [20] and Sanita et al. [21] with modifications.

### Antifungal susceptibility testing

Susceptibilities of the isolates to antifungal drugs were assayed by the broth microdilution method in 96-well plates according to the proposed Clinical and Laboratory Standards Institute M27-A2 and M27-A3 [22, 23].

### Biofilm production assays

The biofilm production assays was previously published by Pierce al. [24] using a rapid reproducible 96-well microtiter-based method. Each strain was performed in triplicate wells. Quantification of biofilm formation was performed at 8 h, 24 h, 48 h and 72 h respectively after inoculation.

### Statistical analysis

The statistical significance of the results was determined by the *t* test and Pearson's chi-squared test, using SPSS version 11.5 (SPSS, Inc., Chicago, IL). The results were considered statistically significant with *P* values of less than 0.05.

## Results

The clinical characteristics of VVC caused by *C. africana* were shown on Table 1. All the patients were symptomatic. Mycological cure rate of VVC caused by *C. africana* was 97% (33/34 cases) and 91% (31/34 cases) at day 7–14 and day 30–35 follow-up when treated with commonly used antifungal agents (Table 1).

**Table 1:** Demographic and clinical characteristics of 34 patients with vulvovaginal candidiasis caused by *C. africana*

Characteristics	*n* (%)
Mean age (years old)	31.15 ± 7.58
Duration (months)	33.97 ± 127.27
Antibiotics (in prior 6 weeks, oral or vaginal)	2(5.9%)
Antifungal on or recent (prior 6 weeks)	4(11.8)
Symptoms and signs	
Pruritis	32(94.2)
Soreness	13(38.3)
Discharge	30(5.9)
Erythema	26(76.5)
Therapy outcome	
7–14 days follow up(Negative)	33(97.1)
30–35 days fillow up(Negative)	31(91.2)

**Fig. 1:**
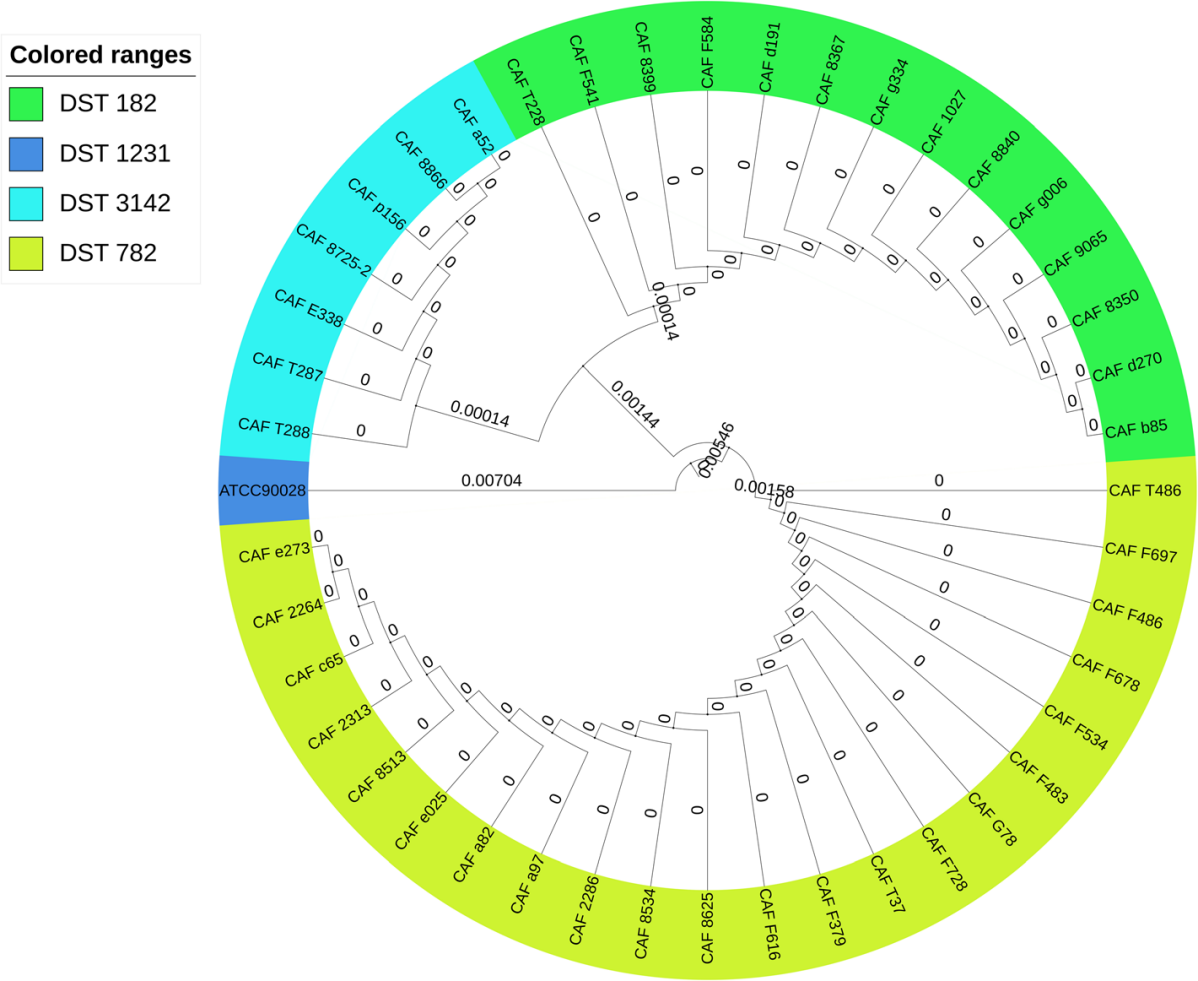
UPGMA dendrogram of 43 *C. africana* strains based on the seven loci used in the MLST analysis and DSTs assigned by *C. albicans* MLST database. 43 *C. africana* strains were divided into 1 clade in the UPGMA dendrogram. DST782 and DST182 were the main MLST types of *C. africana*

The MLST analysis revealed that the DST782 and DST182 were the main MLST genotypes in *C. africana* from vaginal specimens of patients with VVC (Fig. 1; Additional file 2: Table S2).

**Fig. 2:**
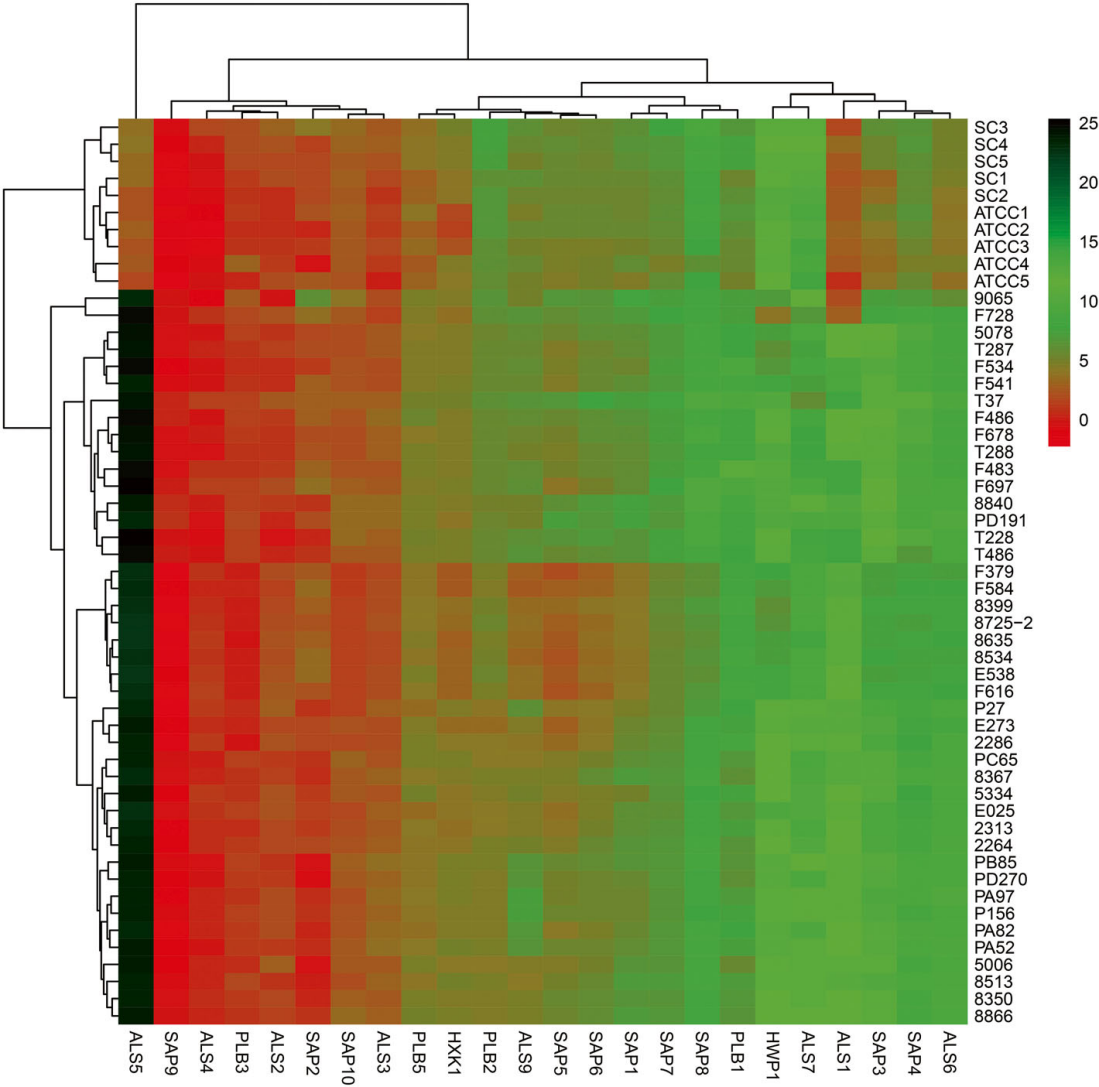
Expression of the virulence genes in *C. africana and C. albicans* by quantitative real-time PCR. Compared with the control strains *C. albicans* ATCC90028 and SC5314*,* the expression of virulent genes ALS1, ALS5, ALS6, SAP3 and SAP4 decreased in *C. africana*

Additional file 8: Figure S3 was electrophoretogram of real time PCR products of virulence genes and drug resistance genes. PCR products of *C. africana* were electrophoresed in agarose gel in TBE buffer and further verified the correct fragment sizes and specificities of primers used in the study. Compared with *C. albicans* SC5314 and *C. albicans* ATCC90028*,* the expression of virulence genes *ALS1*, *ALS5*, *ALS6*, *SAP3* and *SAP4* decreased in *C. africana* strains (Fig. 2; Additional file 4: Table S4)*.*

**Fig. 3:**
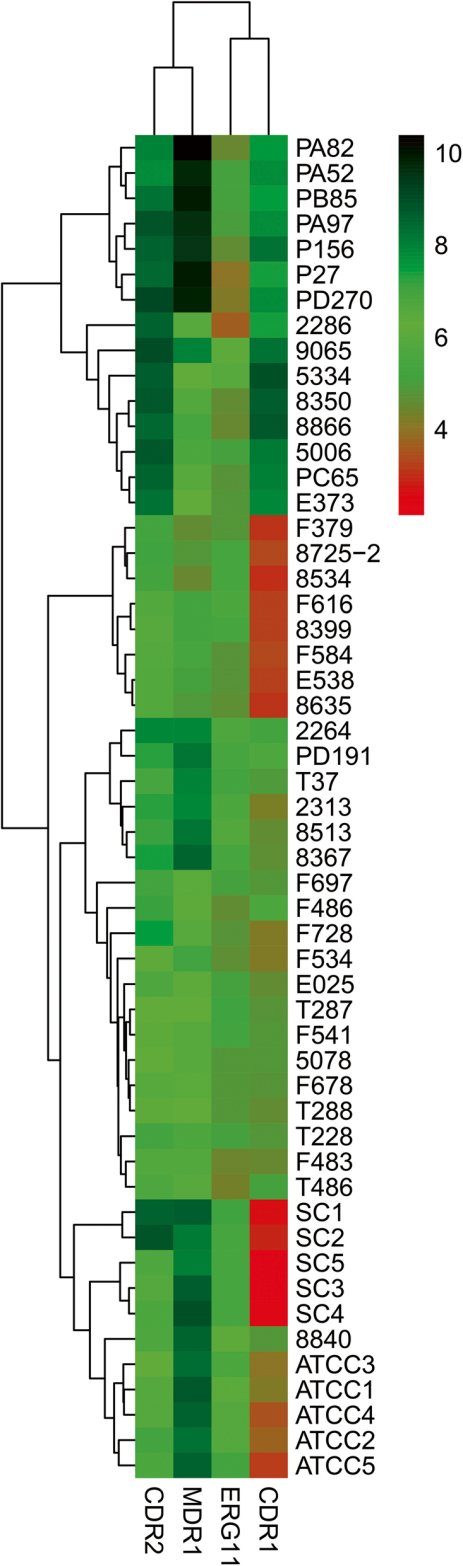
Expression of the drug resistance genes in *C. africana and C. albicans* by quantitative real-time PCR. The expression of CDR1, CDR2, and MDR1 varied among *C. africana*

**Fig. 4:**
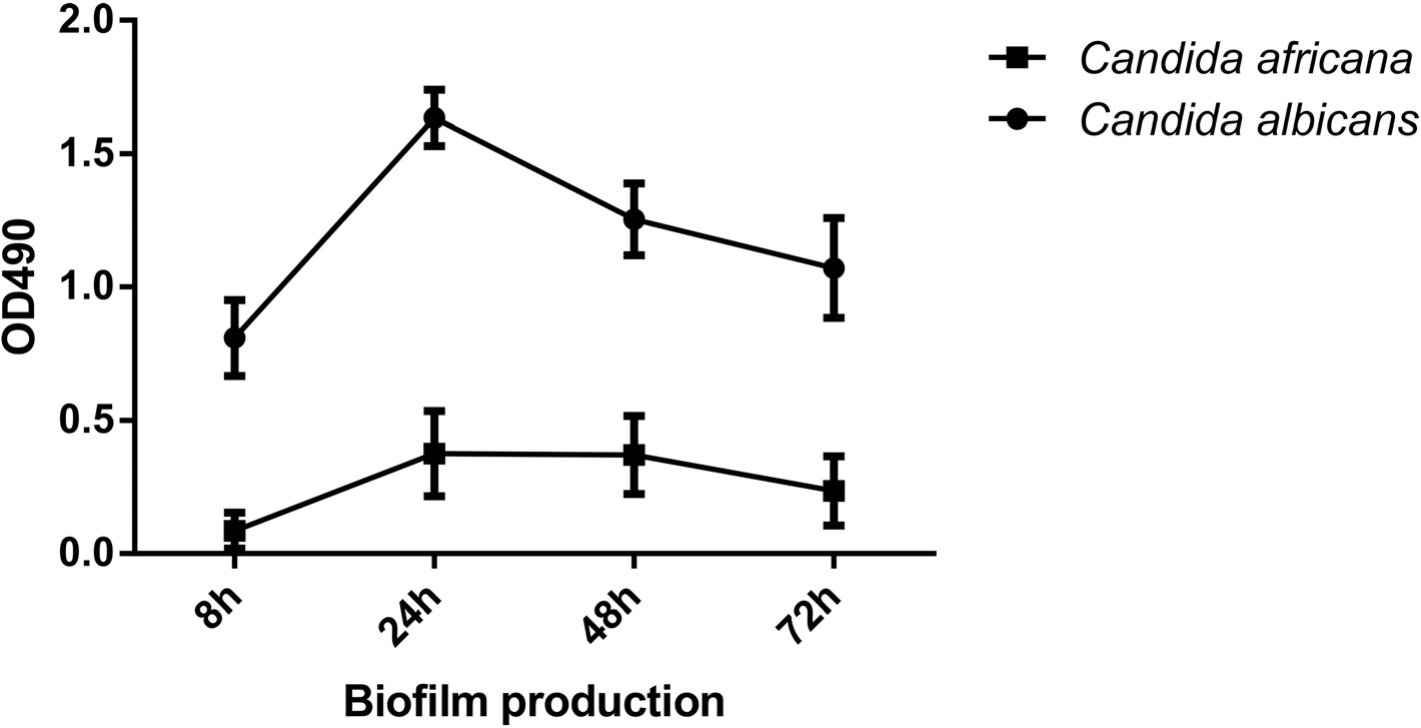
Assessment of biofilms at 8 h, 24 h, 48 h and 72 h at OD490 nm in microtiter plate reader. Biofilm production were significantly lower in *C. africana* than that in *C. albicans*

The expression of drug resistance genes *CDR1*, *CDR2* and *MDR1* varied among strains of *C. africana* (Fig. 3; Additional file 3: Table S3). There were statistically significant differences in biofilm formation and phospholipase activity between *C. africana* and *C. albicans*. Assessment of biofilms at 8 h, 24 h, 48 h and 72 h at OD490 nm in microtiter plate reader showed biofilm production was significantly lower in *C. africana* than that in *C. albicans* (Fig 4).

The sequencing of the partial *HXK1*-gene of *C. africana* strains showed that the region contained the guanine insertion (Additional file 9: Figure S4). All isolates of *C. africana* and control *C. albicans* ATCC90028 and SC5314 displayed positive phospholipase, hemolytic and esterase activities. However, *C. africana* showed less active in phospholipase production (Additional file 7: Figure S2). All *C. africana* strains examined were unable to produce chlamydospores on cornmeal agar plates supplemented with 1% Tween 80 (Qingdao Hopebio-Technology, China) and did not grow in tubes containing GlcNAc as the sole carbon source. All *C. africana* were the genotype A of *C. albicans* and heterozygous [a/α] at the mating-type-like locus.

The mycological cure rate of patients infected with *C. africana* was high when treated with commonly used antifungal agents, which was consistent with in vitro antifungal susceptibilities (Table 2). All *C. africana* isolates were susceptible to the tested antifungal agents (Tables 3 and 4).

**Table 2:** Therapeutic efficacy in patients with VVC caused by *candida africana*

Treatment regimen	Number of cases	Cure cases at follow-up (%)	
		Days 7–14	Days 30–35
Oral fluconazole 150 mg one dose	2	2	2
Oral fluconazole 150 mg two doses	7	6	6
Oral fluconazole 150 mg three doses	2	2	2
Oral itraconazole 200 mg twice daily for one day	2	2	2
Oral itraconazole 200 mg twice daily for two days	4	4	2
Oral itraconazole 200 mg twice daily for five days	1	1	1
Mmiconazole nitrate vaginal suppository 1200 mg one dose	1	1	1
Miconazole nitrate vaginal suppository 1200 mg two doses	4	4	4
Clotrimazole vaginal tablets 500 mg for 1 day	2	2	2
Clotrimazole vaginal tablets 500 mg for 2 days	5	5	5
Terconazole vaginal suppository 80 mg for 6 days	2	2	2
Nystatin vaginal suppository 200,000 IU daily for 7 days	1	1	1
Fluconazole 150 mg two doses together with nystatin vaginal suppository 200,000 IU Daily for 14 days	1	1	1
Total	34	33(97.1)	31(91.2)

**Table 3:** In vitro antifungal susceptibilities of 43 clinical *C. africana* isolates as determined by the Clinical and Laboratory Standards Institute (CLSI) method

Candida species		BUC	CLO	FLC	ITC	MIC	TEC	VRC
*C. africana* DST 182 (14)	Range GM MIC90	0.030–1.00 0.096 0.500	0.030–1.00 0.044 0.030	0.125–2.00 0.540 2.000	0.030–0.03 0.030 0.030	0.015–0.50 0.083 0.500	0.030–0.50 0.047 0.060	0.030–0.25 0.037 0.030
*C. africana* DST 782 (22)	Range GM MIC90	0.015–0.25 0.037 0.125	0.03–0.125 0.032 0.030	0.250–2.00 0.484 1.000	0.015–0.03 0.027 0.030	0.015–0.25 0.024 0.030	0.03–0.125 0.051 0.125	0.030–0.06 0.030 0.030
*C. africana* DST 3142 (7)	Range GM MIC90	0.030–0.50 0.060 0.250	0.030–0.03 0.030 0.030	0.250–0.50 0.410 0.500	0.030–0.03 0.030 0.030	0.015–0.50 0.045 0.500	0.03–0.125 0.044 0.060	0.03–0.125 0.036 0.030
Sub-total (43)	Range GM MIC90	0.015–1.00 0.051 0.250	0.030–1.00 0.034 0.030	0.125–2.00 0.482 1.000	0.015–0.03 0.028 0.030	0.015–0.50 0.036 0.500	0.030–0.50 0.049 0.125	0.030–0.25 0.033 0.030
*C. albicans* ATCC90028 (5)	Range GM MIC90	0.03–0.125 0.039 0.125	0.030–0.06 0.034 0.060	0.500–1.00 0.757 1.000	0.030–0.06 0.052 0.060	0.030–0.50 0.060 0.500	0.030 0.030 0.030	0.030 0.030 0.030

*BUC* butoconazole, *CLO* Clotrimazole, *FLC* Fluconazole, *ITC* Itraconazole, *VRC* Voriconazole, *MIC* Miconazole, *TEC* Terconazole

**Table 4:** In vitro antifungal susceptibilities of 43 clinical C. africana isolates as determined by the Clinical and Laboratory Standards Institute (CLSI) method

Candida species		AmB	FLU	NYS	TEB	AFG	CFG	MFG
*C. africana* DST 182 (14)	Range GM MIC90	0.030–0.250 0.052 0.125	0.250–8.000 1.166 4.000	0.125–4.000 0.734 4.000	8.000–256.000 47.031 128.000	0.008–0.030 0.015 0.015	0.125–0.500 0.396 0.500	0.015–0.500 0.056 0.125
*C. africana* DST 782 (22)	Range GM MIC90	0.030–1.000 0.064 0.250	0.125–2.000 0.567 2.000	0.125–8.000 0.707 4.000	4.000–256.000 21.008 128.000	0.008–2.000 0.013 0.015	0.005–0.500 0.208 0.500	0.015–0.125 0.026 0.125
*C. africana* DST 3142 (7)	Range GM MIC90	0.030–0.125 0.061 0.125	0.125–2.000 0.410 1.000	0.250–4.000 0.371 0.250	4.000–128.000 28.983 64.000	0.008–0.015 0.010 0.015	0.005–0.500 0.192 0.500	0.015–0.125 0.030 0.125
*Sub-total* *(*43)	Range GM MIC90	0.030–1.000 0.060 0.125	0.125–8.000 0.633 2.000	0.125–8.000 0.633 4.000	4.000–256.000 26.979 128.000	0.008–2.000 0.013 0.015	0.005–0.500 0.239 0.500	0.015–0.500 0.032 0.125
*C. albicans* ATCC90028 (5)	Range GM MIC90	1.000 0.375 1.000	0.500–1.000 0.250 1.000	0.250–0.500 0.435 0.500	64.000–256.000 190.74 256.00	0.008–0.015 0.009 0.015	0.250 0.250 0.250	0.008–0.015 0.009 0.015

*AmB* Amphotericin B, *FLU* Flucytosine, *NYS* Nystatin, *TEB* Terbinafine, *AFG* Anidulafungin, *CFG* Caspofungin, *MFG* Micafungin

## Discussion

### MLST and sequence variability

*Candida africana* shows a global distribution and mainly causes genital infections [7]. Review of *C. africana* in literature has been summarized in Additonal file 5: Table S5 and displayed as a world map in Additonal file 10: Figure S5. By using a single pair of primers derived from *HWP1* genes, Romeo and Criseo [13] described an accurate molecular assay for the discrimination among *C. africana*, *C. albicans*, and *C. dubliniensis*. DTS182 was the most common and geographically dispersed strain type having been isolated in Europe, South America, Africa and Asia [6–8]. DST782 isolates were the commonest Asian genotype [6, 7]. All these *C. africana* strains exhibit a phenotypic and genetic homogeneity that is different from their country of origin [7]. In current study, 5 of the 7 MLST loci sequenced showed the same sequences and without single nucleotide polymorphism (SNP) diversity. This suggests a reasonable low level of divergence in the population structure of *C. africana* [7, 10]. The low level of sequence change also suggests that this typing technique may not be ideal for local epidemiological studies [6, 7].

### Phenotypic characterizations, virulence and pathogenicity

Compared to *C. albicans*, *C. africana* shows remarkable phenotypically differences such as the inability to produce characteristic chlamydospores and the incapacity to assimilate many carbon sources especially N-acetylglucosamine(GlcNAc), which plays an important role in cell signaling [1, 5, 25]. DNA sequence analysis of the *HXK1* gene, encoding the enzyme GlcNAc-kinase, demonstrated the existence of a specific mutation [guanine insertion] that impairs GlcNAc assimilation in *C. africana* [19]. All of our *C. africana* carried a specific *HXK1* gene mutation and were mating-type a/α and genotype A of *C. albicans* isolates.

*Candida africana* shows a poor adhesion to human epithelial cells as demonstrated by using both mammalian and insect infection models [2]. In our current study, there were statistically significant differences in biofilm formation, phospholipase activity between *C. africana* and *C. albicans*. All of our patients were symptomatic, which were consistent within vitro extracellular enzymes assays and the expression of virulence genes.

The clinical signs in the patients infected with *C. africana* were almost identical with those with *C. albicans* [9].

### Antifungal susceptibilities and therapy outcome

*Candida africana* exhibits susceptibility patterns of commonly used antifungal agents similar to those of *C. albicans* [8, 9]. However, Al-Hedaithy and Fotedar [26] reported that chlamydospore-negative combined with germ tube negative isolates were resistant to fluconazole and itraconazole. In agreement with previous reports [6, 8, 27], our *C. africana* isolates tested were found to be susceptible to all antifungal agents. Mendling et al. [28] reported that *C. africana* could be eradicated by a single dose vaginal tablet containing 500 mg clotrimazole.

The mycological cure rate of patients infected with *C. africana* was high when treated with commonly used antifungal agents, which was consistent with in vitro antifungal susceptibilities.

## Conclusions

*Candida africana,* a worldwide distribution candida, mainly cause vaginal infections and appears to be with a low level of sequence variation in MLST loci. *Candida africana,* a lower virulence and pathogenicity Candida, is susceptible to commonly used antifungal agents and have a high mycological cure rate.

## Additional files

Additional file 1: **Table S1.** Primers used for amplification, sequencing and expression analysis in this study. (DOC 55 kb)

Additional file 2: **Table S2.** Details of MLST diploid sequence types for 43 *C. africana* strains. (XLSX 14 kb)

Additional file 3: **Table S3.** Expression of the drug resistance genes in *C. africana* and *C. albicans* SC5314 and ATCC90028. (XLSX 12 kb)

Additional file 4: **Table S4.** Expression of the virulence genes in *C. africana* and *C. albicans* SC5314 and ATCC90028. (XLSX 21 kb)

Additional file 5: **Table S5.** Review of *C. africana* in literature. (XLSX 12 kb)

Additional file 6: **Figure S1.** Molecular discrimination of *C. albicans, C. africana*, and *C. dubliniensis* using *HWP1* gene. Lanes 1, 4, 5, 6, 8, and 9 are *C. albicans*; Lanes 7 is *C. africana*. Lanes 10 is *C. dubliniensis* CBS 7988. Lane 2 contains molecular size markers. (JPG 351 kb)

Additional file 7: **Figure S2.** Extracellular enzymatic activity of *C. africana* isolates*.* All isolates of *C. africana* and control *C. albicans* ATCC90028 and SC5314 display positive phospholipase (a, b), hemolytic (c, d) and esterase activities (e, f). *C. africana* shows less active in phospholipase production (a). (JPG 1751 kb)

Additional file 8: **Figure S3.** Real time PCR products of virulence genes and drug resistance genes of *C. africana*. Upper lane 1 to 16 are PCR products of gene *ACT, SAP1, SAP2, SAP3, SAP4, SAP5, SAP6, SAP7, SAP8, SAP9, SAP10, HWP1, PLB1, PLB2, PLB3* and *PLB5*. Lower lane 1 to 13 are PCR products of gene *ACT, ALS1, ALS2, ALS3, ALS4, ALS5, ALS6, ALS7, ALS9, CDR1, CDR2, MDR1* and *ERG11*. Lane M is molecular size marker. (JPG 1187 kb)

Additional file 9: **Figure S4.** Amplification and sequencing of the partial HXK1-gene sequencing of *C. africana* and *C. albicans* ATCC90028. *C. africana* strain is showing the region containing the guanine insertion. (JPG 595 kb)

Additional file 10: **Figure S5.** The world distribution of reported *C. africana* in literature The map was developed by using Adobe Photoshop CS6 (v13.0.1.1, Adobe Systems, San Jose, CA, USA). The copyright holder grants anyone the right to use this work for any purpose, without any conditions, unless such conditions are required by law. https://upload.wikimedia.org/wikipedia/commons/a/a2/2009_Special_301_Report_%28World_Map%29.png. (JPG 3368 kb)

## Abbreviations

ABC: ATP-binding cassette
ALS: Agglutinin-like sequence
CDR: Candida drug resistance
DST: Diploid sequence typing
ERG: Ergosterol
GlcNAc: N-acetylglucosamine
HWP1: Hyphal wall protein 1
HXK1: Hexokinase 1
MDR: Multidrug resistant
MIC: Minimal inhibitory concentration
MLST: Multilocus sequence typing
PCR: Polymerase chain reaction
PLB: Phospholipase B
SAP: Secretory aspartyl proteinase
VVC: Vulvovaginal candidiasis
