# Supplementary material for: Multilocus sequence typing analysis of Candida africana from vulvovaginal candidiasis
Source: BMC Infect Dis. 2019 May 22;19:461. doi: 10.1186/s12879-019-4071-7 (PMC6532261; doi:10.1186/s12879-019-4071-7)
Supplement: Supplementary file 1 — Table S1. Primers used for amplification, sequencing and expression analysis in this study. (DOC 55 kb) [file 12879_2019_4071_MOESM1_ESM.doc]

Table S1 Primers used for amplification, sequencing and expression analysis

| **Fragement** | **Forward(5’-3’)** | **Reverse(5’-3’)** | **References** |
| --- | --- | --- | --- |
| HWP1 | GCTACCACTTCAGAATCATCATC | GCACCTTCAGTCGTAGAGACG | Romeo and Criseo.(2008) |
| CA-INT | ATA AGG GAA GTC GGC AAA ATA GAT CCGTAA | CCT TGG CTG TGG TTT CGC TAG ATA GTAGAT | McCullough et al. (1999) |
| AAT1a | ACTCAAGCTAGATTTTTGGC | CAGCAACATGATTAGCCC | Bougnoux et al.(2003) |
| ACC1 | GCAAGAGAAATTTTAATTCAATG | TTCATCAACATCATCCAAGTG | Bougnoux et al.(2003) |
| ADP1 | GAGCCAAGTATGAATGATTTG | TTGATCAACAAACCCGATAAT | Bougnoux et al.(2003) |
| MPIb | ACCAGAAATGGCCATTGC | GCAGCCATGCATTCAATTAT | Bougnoux et al.(2003) |
| SYA1 | AGAAGAATTGTTGCTGTTACTG | GTTACCTTTACCACCAGCTTT | Bougnoux et al.(2003) |
| VPS13 | TCGTTGAGAGATATTCGACTT | ACGGATGGATCTCCAGTCC | Bougnoux et al.(2003) |
| ZWF1b | GTTTCATTTGATCCTGAAGC | GCCATTGATAAGTACCTGGAT | Bougnoux et al.(2003) |
| HXK1 | GACTAGCATTAGTGGGTTGCG | CACCCAGCAGAATACACCG | Felice et al. (2016) |
| HXK1-RT | CAAATTGTTGGGCAGTGACG | TGATAGGCAGCACCTATGGC | Felice et al. (2016) |
| ACT1 | TGCTGAACGTATGCAAAAGG | TGAACAATGGATGGACCAGA | Alves et al.(2014) |
| SAP1 | TGAGGCTGCTGGTGATTATG | TGCCAACAGCTTTGAGAGAA | Correia et al.(2010) |
| SAP2 | ATCAGCTGGTTTCGTTGCTT | GGGACAGCTTGTCTTTTGGA | Correia et al.(2010) |
| SAP3 | TGTTACTGGTCCCCAAGGTGAA | CTTGTCCTTGACCAGCTTGACAT | Correia et al.(2010) |
| SAP4 | AATGATGTGGGCAAAAGAGG | ACGGCATTTGAATCTGGAAC | Correia et al.(2010) |
| SAP5 | ATTTCCCGTCGATGAGACTG | ACCACGCCATTTTGGAATAC | Correia et al.(2010) |
| SAP6 | GTCAACGCTGGTGTCCTCTT | GCAGGAACGGAGATCTTGAG | Correia et al.(2010) |
| SAP7 | TTCTCGTGATGCTGTCCAAG | CCAGCAGGAAGACCATAAGC | Correia et al.(2010) |
| SAP8 | TTTGGTGGGGTTGATAATGC | GGCAGCAGCCAATTTATCAG | Correia et al.(2010) |
| SAP9 | ACCGGGTCTTCAGATTTGTG | TTCCTCGTCGGTTTCTATGG | Correia et al.(2010) |
| SAP10 | AACGGAAATGTTGCTTCTGG | TGAATCGCCTATCGAAAACC | Correia et al.(2010) |
| PLB1 | GCTCTTTTCAACGAAGCGGTGT | GCCATCTTCTCCACCGTCAACT | Alves et al.(2014) |
| PLB2 | CAATACTAGCCGCGTTGGGAAG | GCCCATGAAAAACCCTGCATTA | Alves et al.(2014) |
| PLB3 | TCCCAATTGTTGTTGCTGATGG | CCGCATTATCAAACCCACCAAT | Alves et al.(2014) |
| PLB5 | TCGTCCGGGTCTTCAAGTTCTC | ATCTCCCGAATCCCCGTCTAAA | Alves et al.(2014) |
| ALS1 | CCCAACTTGGAATGCTGTTT | TTTCAAAGCGTCGTTCACAG | Alves et al.(2014) |
| ALS2 | GCACTTCATTGACTGGAGCA | TCATTGTTGCCACCTTGTGT | Alves et al.(2014) |
| ALS3 | CTGGACCACCAGGAAACACT | GGTGGAGCGGTGACAGTAGT | Alves et al.(2014) |
| ALS4 | TCCACAGTTTCTCGTCCACA | ATTGCCACGCTTGTTTTACC | Alves et al.(2014) |
| ALS5 | GTTCAGACATGCCATCATCG | CCAAGTGATCAGGGTGGACT | Alves et al.(2014) |
| ALS6 | ATCGGAAGCTCCAAATTCCT | AGGATGTTTAGTGGCGGATG | Alves et al.(2014) |
| ALS7 | GACCTTTTGTGGATGCGATT | TTTTCTGGAGTCGGGAAATG | Alves et al.(2014) |
| ALS9 | CCATATTCAGAAACAAAGGGTTC | AACTGAAACTGCTGGATTTGG | Green et al.(2004) |
| HWP1-RT | TCTACTGCTCCAGCCACTGA | CCAGCAGGAATTGTTTCCAT | Alves et al.(2014) |
| CDR1 | TTTAGCCAGAACTTTCACTCATGATT | TATTTATTTCTTCATGTTCATATGGATTGA | Chau et al.(2004) |
| CDR2 | GGTATTGGCTGGTCCTAATGTGA | GCTTGAATCAAATAAGTGAATGGATTAC | Chau et al.(2004) |
| MDR1 | TTACCTGAAACTTTTGGCAAAACA | ACTTGTGATTCTGTCGTTACCG | Chau et al.(2004) |
| ERG11 | AACTACTTTTGTTTATAATTTAAGATGGACTATTGA | AATGATTTCTGCTGGTTCAGTAGGT | Chau et al.(2004) |
