# Supplementary figures and images for: Multilocus sequence typing analysis of Candida africana from vulvovaginal candidiasis
Source: BMC Infect Dis. 2019 May 22;19:461. doi: 10.1186/s12879-019-4071-7 (PMC6532261; doi:10.1186/s12879-019-4071-7)

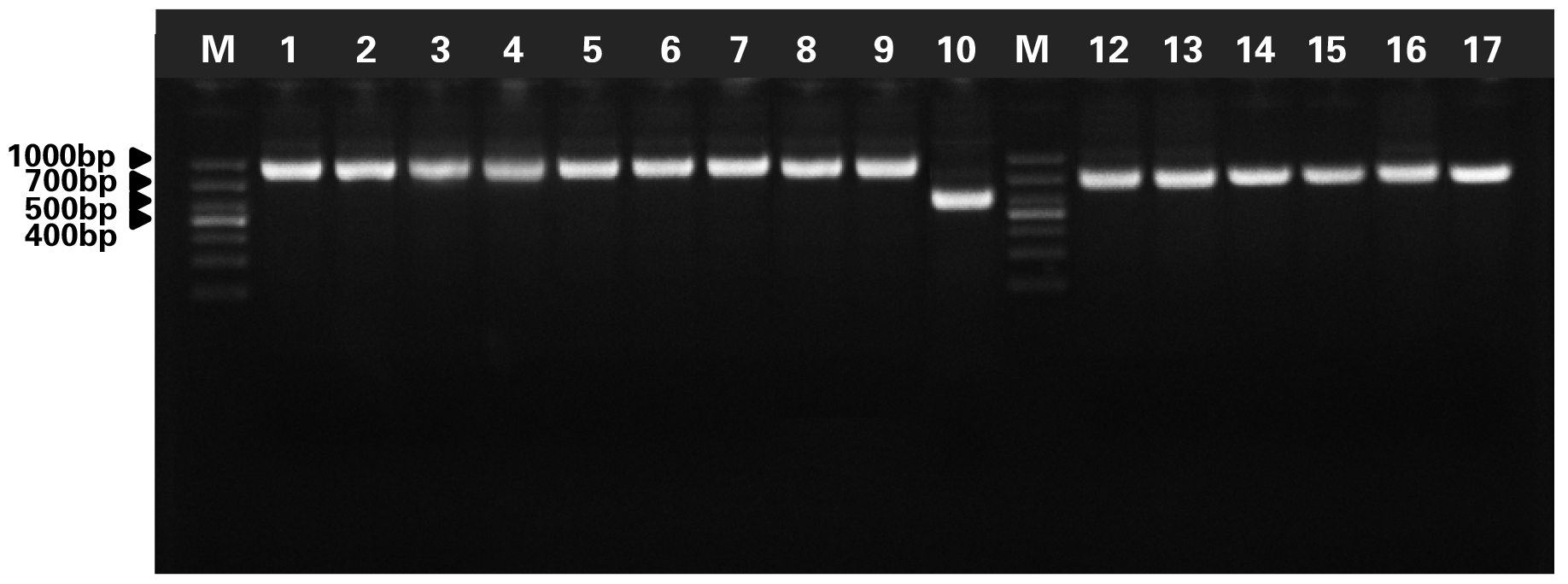

Supplement: Supplementary file 6 — Figure S1. Molecular discrimination of C. albicans, C. africana, and C. dubliniensis using HWP1 gene. Lanes 1, 4, 5, 6, 8, and 9 are C. albicans; Lanes 7 is C. africana. Lanes 10 is C. dubliniensis CBS 7988. Lane 2 contains molecular size markers. (JPG 351 kb) [file 12879_2019_4071_MOESM6_ESM.jpg]

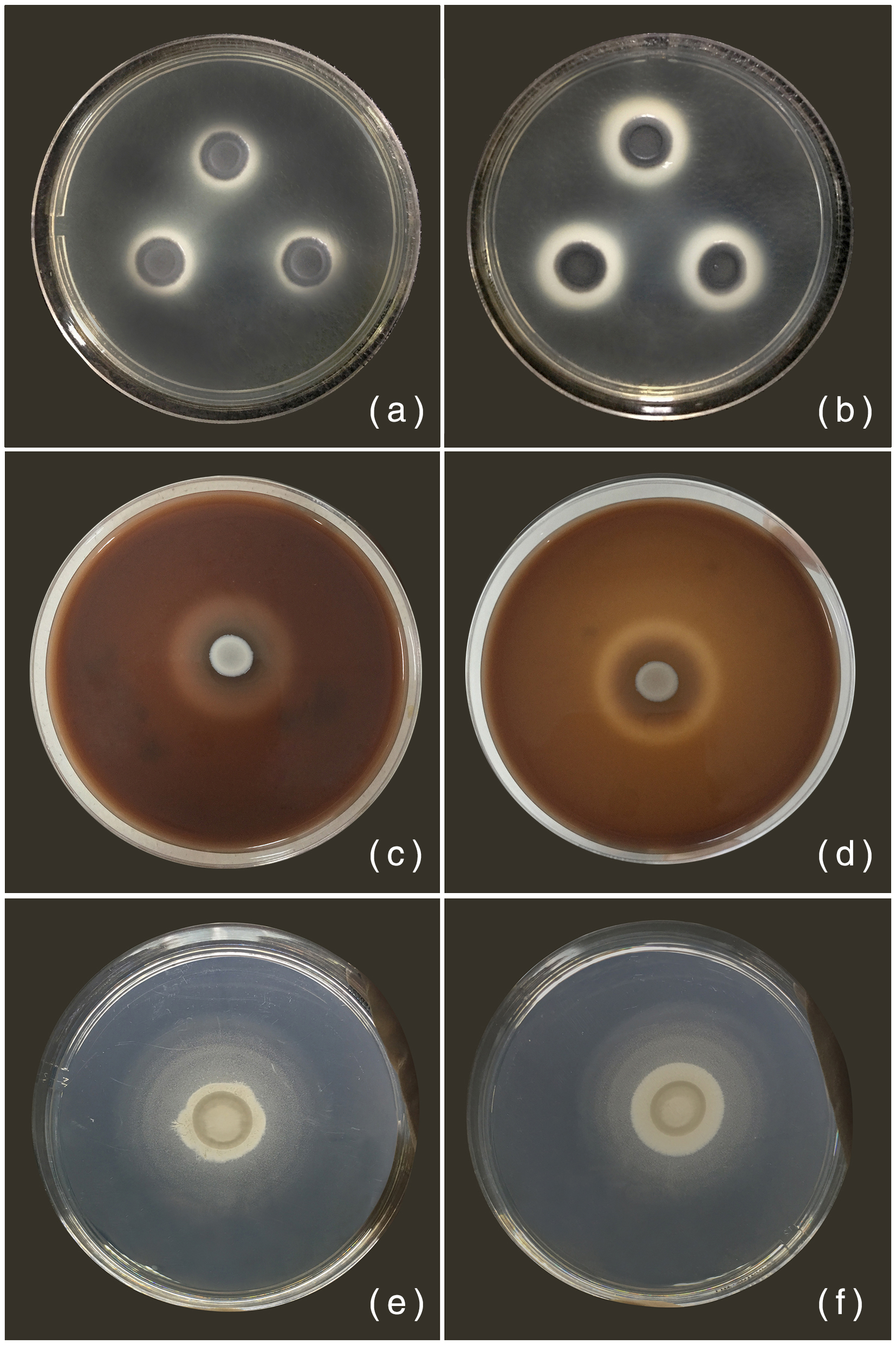

Supplement: Supplementary file 7 — Figure S2. Extracellular enzymatic activity of C. africana isolates. All isolates of C. africana and control C. albicans ATCC90028 and SC5314 display positive phospholipase (a, b), hemolytic (c, d) and esterase activities (e, f). C. africana shows less active in phospholipase production (a). (JPG 1751 kb) [file 12879_2019_4071_MOESM7_ESM.jpg]

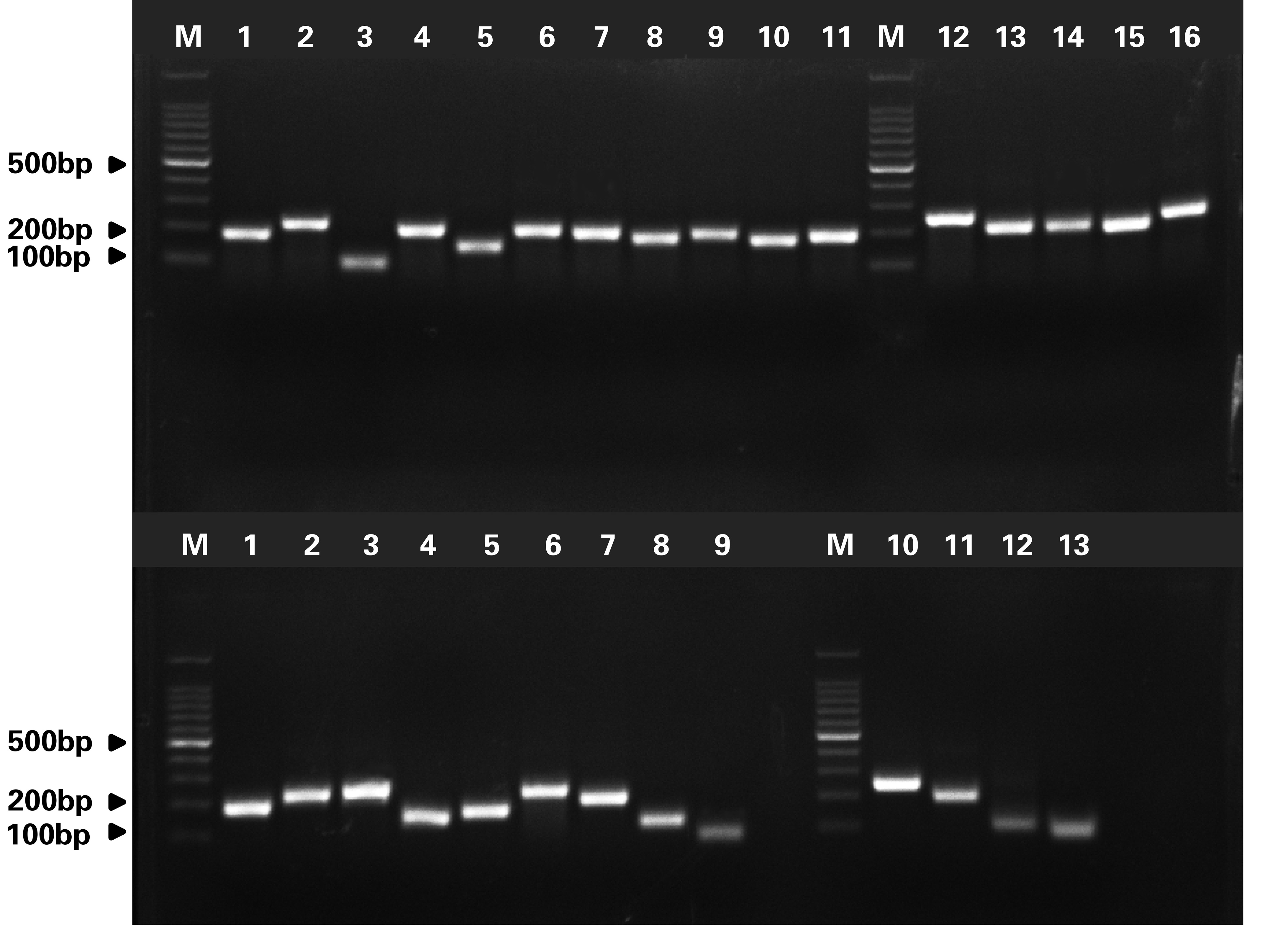

Supplement: Supplementary file 8 — Figure S3. Real time PCR products of virulence genes and drug resistance genes of C. africana. Upper lane 1 to 16 are PCR products of gene ACT, SAP1, SAP2, SAP3, SAP4, SAP5, SAP6, SAP7, SAP8, SAP9, SAP10, HWP1, PLB1, PLB2, PLB3 and PLB5. Lower lane 1 to 13 are PCR products of gene ACT, ALS1, ALS2, ALS3, ALS4, ALS5, ALS6, ALS7, ALS9, CDR1, CDR2, MDR1 and ERG11. Lane M is molecular size marker. (JPG 1187 kb) [file 12879_2019_4071_MOESM8_ESM.jpg]

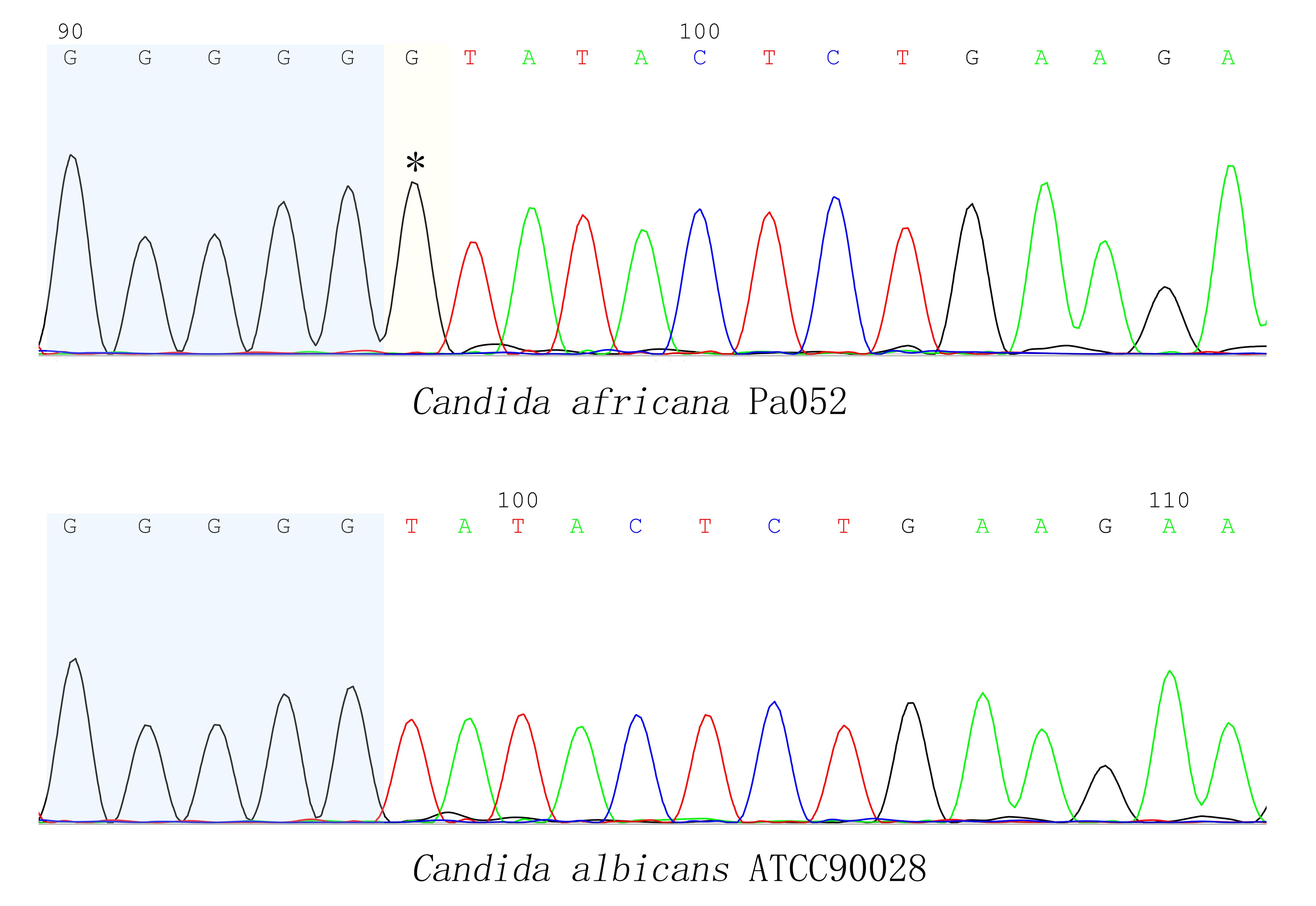

Supplement: Supplementary file 9 — Figure S4. Amplification and sequencing of the partial HXK1-gene sequencing of C. africana and C. albicans ATCC90028. C. africana strain is showing the region containing the guanine insertion. (JPG 595 kb) [file 12879_2019_4071_MOESM9_ESM.jpg]

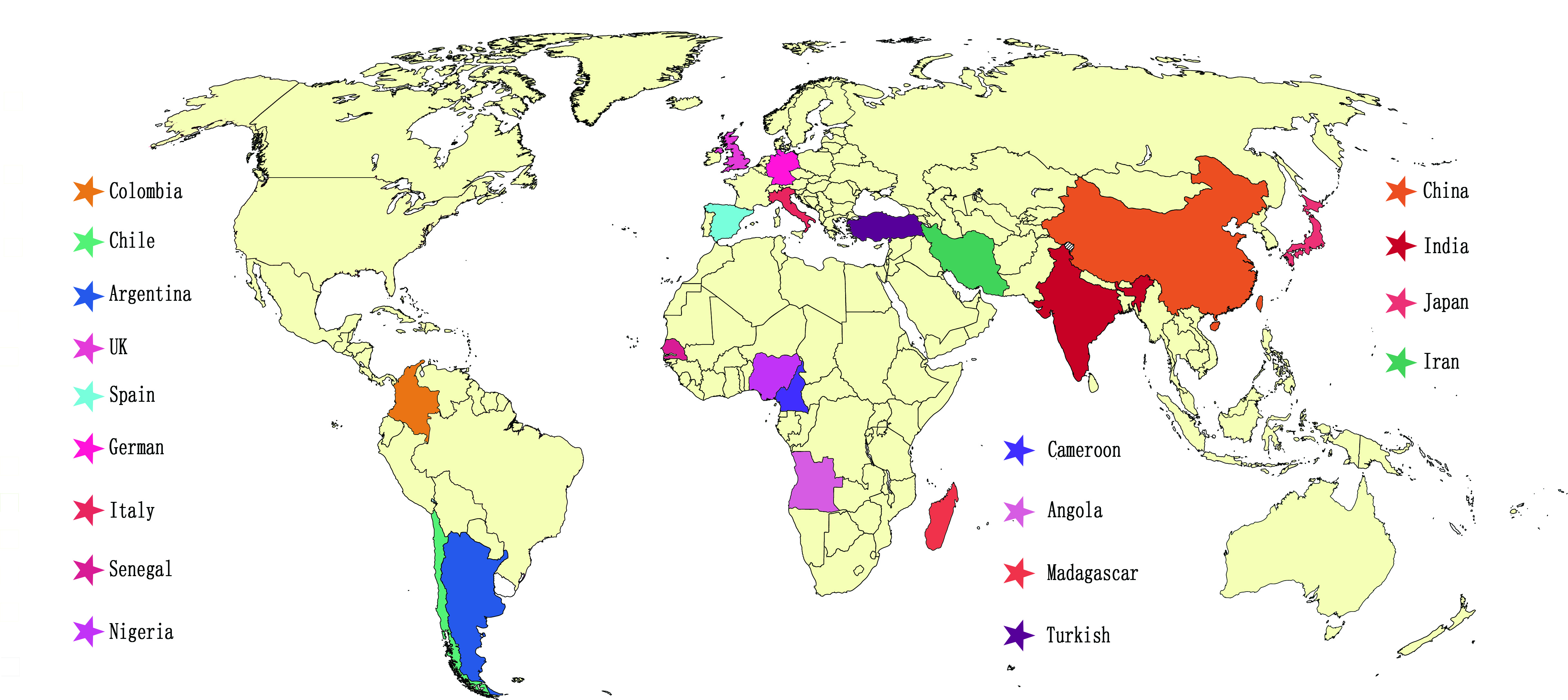

Supplement: Supplementary file 10 — Figure S5. The world distribution of reported C. africana in literature The map was developed by using Adobe Photoshop CS6 (v13.0.1.1, Adobe Systems, San Jose, CA, USA). The copyright holder grants anyone the right to use this work for any purpose, without any conditions, unless such conditions are required by law. https://upload.wikimedia.org/wikipedia/commons/a/a2/2009_Special_301_Report_%28World_Map%29.png. (JPG 3368 kb) [file 12879_2019_4071_MOESM10_ESM.jpg]
